# Access to Resources Shapes Maternal Decision Making: Evidence from a Factorial Vignette Experiment

**DOI:** 10.1371/journal.pone.0075539

**Published:** 2013-09-17

**Authors:** Geoff Kushnick

**Affiliations:** Department of Anthropology and Southeast Asia Center, Henry M. Jackson School of International Studies, University of Washington, Seattle, Washington, United States of America; Birkbeck, University of London, United Kingdom

## Abstract

The central assumption of behavioral ecology is that natural selection has shaped individuals with the capacity to make decisions that balance the fitness costs and benefits of behavior. A number of factors shape the fitness costs and benefits of maternal care, but we lack a clear understanding how they, taken together, play a role in the decision-making process. In animal studies, the use of experimental methods has allowed for the tight control of these factors. Standard experimentation is inappropriate in human behavioral ecology, but vignette experiments may solve the problem. I used a confounded factorial vignette experiment to gather 640 third-party judgments about the maternal care decisions of hypothetical women and their children from 40 female karo Batak respondents in rural Indonesia. This allowed me to test hypotheses derived from parental investment theory about the relative importance of five binary factors in shaping maternal care decisions with regard to two distinct scenarios. As predicted, access to resources—measured as the ability of a woman to provide food for her children—led to increased care. A handful of other factors conformed to prediction, but they were inconsistent across scenarios. The results suggest that mothers may use simple heuristics, rather than a full accounting for costs and benefits, to make decisions about maternal care. Vignettes have become a standard tool for studying decision making, but have made only modest inroads to evolutionarily informed studies of human behavior.

## Introduction

Evolutionary studies of maternal decision making in humans have met with a mixed bag of successes and setbacks. Although human behavioral ecologists have tested a wide range of hypotheses derived from parental investment theory (see Box 3 in [1]), many of these studies find a lack of support for the predictions of optimality models [2]. One challenge has been the difficulty of disentangling the relative contributions of the variables that shape the fitness costs and benefits of care [3,4].

Behavioral ecology is the evolutionarily guided study of decision-making [5]. Its central concern is how natural selection shapes decision rules—i.e., the ability to adjust behavior facultatively in response to environmental conditions in a way that maximizes inclusive fitness. In the most general sense, decision rules (also known as “adaptive strategies” and “rules of thumb”) take the following form: “in context X, do α; in context Y switch to β” [1]. It might be assumed that conscious choice is necessary because the study of decision rules is steeped in economic jargon. This need not be the case, however, as natural selection can create decision-making abilities that employ sensory, endocrine, neural, and cognitive mechanisms [5]. Behavioral ecologists study decision rules by testing hypotheses derived from optimality models with data on actual or reported behavior [1]. Although human adaptations are shaped by the evolution of genes and culture [6], it is still a reasonable hypothesis that their decision rules should oftentimes lead to outcomes that maximize fitness (cf. [7]). Nonetheless, the study of human decision making has been severely hamstrung by the use of correlational methods over experimental ones, as the tight controls offered by experiments have been crucial in studying the decision rules of other organisms [8-10].

Here, I report the use of a third-party confounded factorial vignette experiment to investigate the role of five factors—mother’s age and access to resources, and child’s age, gender, and viability—in shaping decisions related to maternal care among Karo Batak agriculturalists from rural Indonesia. The results suggest that mothers make decisions using simple heuristics of varying complexity rather than decision rules that account for the full range of costs and benefits. I discuss alternative explanations for the results, as well as potential directions for further study.

### Vignette Experiments

Since their first use in the 1970s [11,12], vignette studies have become a standard tool for studying decision making [13]. Respondents are presented with vignettes designed to “elicit their beliefs, attitudes, judgments, knowledge, or intended behavior with respect to the presented vignette scenarios” where a vignette is a “short, carefully constructed description of a person, object, or situation” [13]. By using experimental designs and systematically varying factors, vignette experiments allow for the estimation of the effects of independent variables without the confounding effect of other variables [11,13]. Factorial designs [14] allow for the estimation of the effects of multiple factors and their interactions. Full factorial designs present each respondent with every possible vignette, but grow exponentially with added factors—e.g., two binary factors yields 4 vignettes (i.e., 2^2^=4), but a single binary and three four-level factors yields 128 (i.e., 2^1^+4^3^=128). When the number of combinations becomes too large for a single respondent, designs that present a subset of the total can be used. The drawback is the loss of ability to estimate accurately some effects [13]. In one such design, the confounded factorial design, the entire vignette population is used but is split into orthogonal vignette sets. This allows for the main effect of each factor and their first-order interactions to be estimated at the expense of the ability to estimate higher-order interaction effects, as they are confounded with set effects [13].

Factorial experiments are well suited for studying systems with complex causation, such as when multiple factors interact with each other [8,10]. When used with vignette methods, they allow the researcher to explore scenarios of varying realism that might not be naturalistically available at the time a survey is administered. For example, the researcher might want to know more about someone with a particular combination of characteristics only to find that no one is available that fits the bill. Without the vignette methodology, it would be impossible to get the probable decisions made by, for instance, a 35-year old food-insecure woman with an unhealthy 24-month-old son if that woman did not exist in the population at the time of study. This is often the case in small-scale, anthropological populations [15]. Further, vignette studies are well suited for testing hypotheses cross-culturally. One potential objection to vignette studies is that they lack ecological validity. This has been countered in studies that have shown tight concordance between responses to vignettes and actual behavior [16]. Despite their benefits, vignette experiments have made only modest inroads to evolutionarily informed studies of human behavior. An electronic keyword-and-text scan (see Table S1 in the *SI*) revealed that only 16 papers published between 1997 and 2012 in the flagship journal of the Human Behavior and Evolution Society, *Evolution and Human Behavior*, used vignettes as their primary or secondary methodology; only eight were studies of decision rules that used vignettes.

## Hypotheses

### Basic Hypothesis/Predictions

The basic hypothesis tested here is derived from parental investment theory [17-19]—that natural selection has shaped the decision-making facilities of human females to optimize the tradeoff between investment in current versus future offspring, or current offspring and other components of maternal fitness. Factors that increase the benefits of investing in current offspring should lead to increased care; factors that increase the costs of care, by decreasing the ability to invest in future offspring or other components of fitness, should lead to decreased care. From this, the following specific predictions about maternal investment decision rules can be derived vis-à-vis the five factors of interest.

#### Mother’s Age

Older mothers are more likely to invest in current offspring because they have lower residual reproductive value, decreasing the cost of care [20]. Support for this effect on maternal investment decisions have been found in studies of age-specific abortion rates in Sweden [21] and gestation lengths in Spain [22]. Using the same logic, Clutton-Brock [23] proposed the terminal investment hypothesis, predicting that mothers in iteroparous species should provide increased care to their final offspring because there is no cost to future reproduction. Support for this effect in humans is mixed [24-26]. Mother’s age may interact with offspring gender and viability. I test the following predictions/decision rules:

•Increase care as you get older (main effect).

•Increase care in daughters if you are young (interaction effect).

•Increase care in sons if you are young (interaction effect).

•Increase care in sickly offspring, but only if you are young (interaction effect).

#### Resource Access

Better access to resources leads to a smaller opportunity cost for investing in current offspring, and thus a greater likelihood of providing care [17,27]. Low [28-30] discusses the influence of resources access on reproductive decision making. Although, the resource in question may be wealth in market-based economies [31-33], other aspects of resource access—such as food availability—may influence female reproductive decisions [34]. Resource access is a component of maternal condition and, thus, may interact with gender of child [35]. I test the following predictions/decision rules:

•Increase care if you have good access to resources (main effect).

•Increase care in sons if you have good access to resources (interaction effect).

#### Child’s Gender

Mothers should invest more in offspring of the gender with greater potential for enhancing their inclusive fitness, which should be moderated by aspects of maternal condition such as her age and access to resources. The Trivers-Willard [35] hypothesis predicts that mothers in good condition should invest more heavily in sons. Cronk [36] reviewed the mixed evidence for this effect in humans. Because early-born children may be recruited as “helpers at the nest” [37], however, younger women may favor their daughters. Quinlan et al. [38] present evidence for local resource enhancement in an analysis of breastfeeding in Dominica. On the other hand, mothers should invest less in offspring that compete with their siblings for resources [39]. In an earlier paper [40], I found that later born sons were treated as “surplus” among the Karo Batak because they competed with their older siblings for inheriting their parents’ farmland. A similar effect was documented in historical Germany [41]. I test the following predictions/decision rules:

•Increase care in sons if you have good access to resources (interaction effect).

•Increase care in daughters if you are young (interaction effect).

•Increase care in sons if you are young (interaction effect).

#### Child’s Age

This factor affects the fitness benefits of care. Younger offspring have greater “need” and thus mothers should be more likely to provide care to them. My previous research among the Karo Batak has provided some support to this hypothesis [42]. Nonetheless, there is some concern that parents, in some circumstances, may favor older offspring because of their higher reproductive value [17,43]. I test the following predictions/decision rules:

•Increase care in younger offspring (main effect).

•Increase care in older offspring (main effect).

#### Child’s Viability

A child’s health status affects the fitness benefits of providing care and, thus, mothers should be very sensitive to cues of offspring health [44]. Sickly offspring will receive higher levels of maternal care when it can enhance health status [45], lower when the offspring has little chance of survival or future reproduction [46,47]. This effect may be moderated by maternal age—i.e., younger mothers should be less likely to provide care to sickly offspring because producing a replacement offspring may have a greater positive effect on their inclusive fitness. I test the following predictions/decision rules:

•Increase care if offspring is sickly (main effect).

•Increase care if offspring is rarely sick (main effect).

•Increase care in sickly offspring, but only if you are young (interaction effect).

### Secondary Hypothesis

The secondary hypothesis is that judgments about the probable maternal care decisions of a hypothetical woman (i.e., the “third-party” perspective) will be reasonable approximations of actual decision rules. This perspective was chosen to circumvent the tendency of parents of parents to provide erroneous self-reports about their own parenting decisions [48]. If the third-party judgments reflect the actual decisions of women like those in the vignettes, and not those of the respondents themselves, the judgments of old and young respondents should not differ.

## Methods

### Ethics Statement

Approval for this project and all of the protocols documented below was granted by the Institutional Review Board at the University of Washington, Seattle. Respondents gave voluntary consent orally before being allowed to participate in the study. Because participants’ names were not recorded on the datasheets, it was deemed that documenting consent would create an unnecessary link between the respondents’ names and responses. Research permits to conduct fieldwork in Indonesia were issued by the Ministry of Research and Technology of the Republic of Indonesia in Jakarta, and at all of the appropriate local levels of government. The University of North Sumatra in Medan served as the local counterpart.

### Design

**Figure 1:**
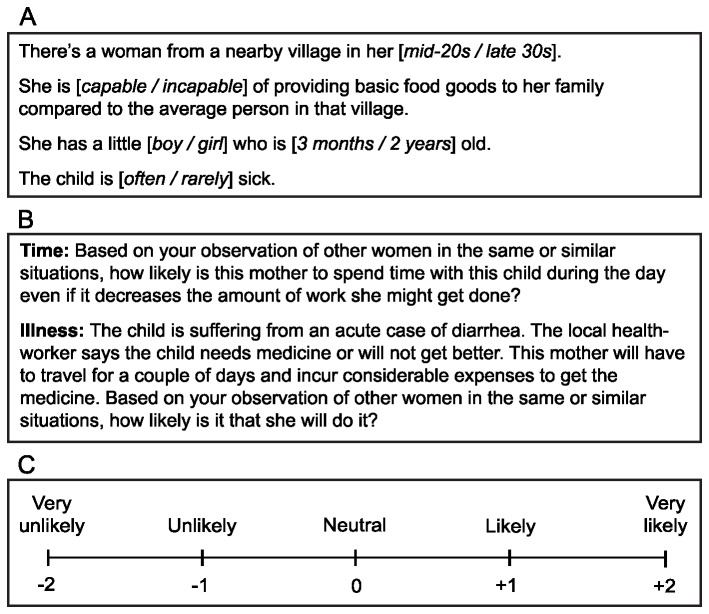
Study design elements. The (A) vignettes, (B) questions, and (C) response scale used in the study. The text of these elements was translated to Bahasa Indonesia before presentation to respondents. The numbers on the response scale were used for analysis, but were not shown to respondents.

I used a confounded factorial vignette experiment [11,13], to elicit third-party judgments about maternal care decisions made by hypothetical women with regards to hypothetical children. I present the wording of the vignettes in Figure 1A. There were 32 total vignettes, constructed by taking all possible combinations of five binary factors (2^5^=32). Presenting the entire population of vignettes to each respondent would have caused fatigue, so I presented each participant with just eight vignettes. I created the vignette sets using the design-of-experiments tools in the software package JMP [49]. Using the software’s terminology, this was done by specifying a custom design with eight blocks (i.e., sets) and 64 runs (i.e., so each set had eight vignettes) that would allow for the unconfounded estimation of all main and first-order interaction effects. I used eight sets instead of four so that the vignettes could be presented in two varied orders. The details of each vignette set are included in the *SI* (see Table S2).

Levels for each factor in the vignettes were as follows: *Mother’s age* (mid-20s or late 30s) provided a contrast between mothers near the beginning and end of their reproductive spans; *Resource access*, measured as mother’s ability to provide food for her children (capable or not capable) provided a contrast that would be easily translated to any society; *Child’s gender* (male or female); *Child’s age* (3 months or 2 years old) provided a contrast between a child who most likely would still be breastfeeding and one who was close to weaning age [42], but both children would still be highly dependent on their mothers; and, *Child’s viability* (often sick or rarely sick) provided a contrast between a child who is sickly and one who is not.

The dependent variables were judgments of how likely the hypothetical woman was to engage in the behavior described in each question, which are included word for word in Figure 1B. I wrote the questions to provide scenarios with contrasting ramifications. In the Time question, the decision was whether to spend time with offspring if it came at the expense of economic productivity. In the *Illness* question, the decision was whether to incur a substantial expense of time and resources to get treatment for a gravely ill child. Responses were measured on a five-point scale, as shown in Figure 1C.

### Participants

I collected judgments (*n*=640) from 40 Karo Batak women from two villages. Stratified random sampling was used to ensure that women from a broad range of ages were included: 18-25, 26-35, 36-45, and 46+ years old. Only ever-married, non-nulliparous women were allowed to participate to ensure that those women who, for whatever reason, might be disinterested in the childrearing process were excluded from the sample. This was necessary because the design centered on third-party judgments.

The Karo Batak are a group of patrilineal agriculturalists from North Sumatra, Indonesia [50], among whom I have conducted almost 24 months of fieldwork. Village of residence was included in the analyses because there are substantive inter-village ecological differences [51], as well as differences in patterns of childbearing and childrearing [40]. The first village, Doulu (3°13′23″ N, 98°31′50″ E), sits at 915 m above sea level. It has a relatively higher fertility rate (4.3 live births per woman) and its sub-regency has a relatively lighter disease load (243 diarrhea and 0 malaria cases per 10,000 individuals per year) [51]. The second, Laubuluh (3°10′49″ N, 98°16′12″ E), sits at 762 m above sea level. It has a relatively lower fertility rate (3.5 live births per woman) and its sub-regency has a relatively heavier disease load (621 diarrhea and 293 malaria cases per 10,000 individuals per year) [51].

### Procedure

After obtaining consent, interviews were conducted in private by a trained female research assistant for approximately 30 minutes to 1 hour. Participants were reimbursed 5,000rp (approximately $0.60) for their time. Participants were cycled through the vignette sets. That is, the first participant was assigned Set A, the second Set B, and so on. The assistant presented the vignettes in the order listed in Table S2 for the appropriate vignette set (see *SI*). Vignettes were read aloud to the participant, who answered by pointing to the response card with the five-point scale written on it as in Figure 1C, but not the numerical values. If the response was midway between two points, the assistant rounded the answer up.

### Statistical Analyses

Analyses were conducted using linear random-intercept models to adjust for multiple judgments from each respondent [52]. Reported models included all main effects, the first-order interaction effects necessary for hypothesis testing, and a series of dummy variables to control for set effects—i.e., effects caused by being assigned to a certain vignette set. Full models, labeled Model X, were estimated using all judgments from all respondents, and included terms for respondent age in years and village. Models Y_1_ and Y_2_, were estimated using the younger (<35 years old) and older (≥ 35 years old) halves of the sample, and included a term for village. I conducted all analyses in Stata 10.1. I have included the raw data in the *SI* (see Table S3).

## Results

### Time

**Figure 2:**
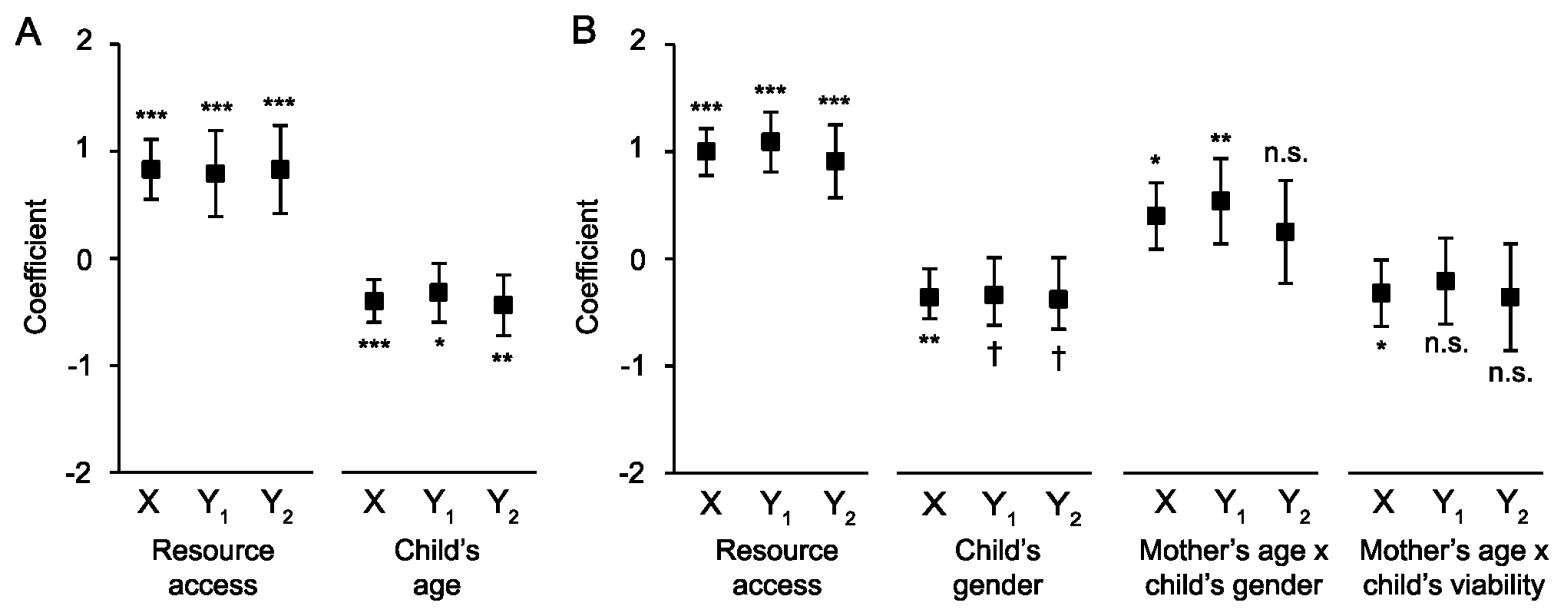
Coefficients from the linear random-intercept models. Statistically significant coefficients from the linear random-intercept models for the (A) Time and (B) *Illness*. Error bars are 95% confidence intervals. Factors that were not statistically significant are not illustrated, but those that were statistically significant in Model X, but not Model Y_1_ or Y_2_, are included. Sample sizes: Model X, *n*=320; Models Y_1_ and Y_2_, *n*=160 each. Significance: * *p*<0.05, ** *p*<0.01, *** *p*<0.001, † *p*<0.10.

I used a linear random-intercept model to estimate the effect of each factor and the interactions of interest on responses to the Time question. This provided an adjustment for having multiple judgments per respondent. The significant coefficients are illustrated in Figure 2A. The full details of the models are included in the *SI* (see Table S4). Model X was estimated using all judgments from all respondents, and there were two substantively and statistically significant main effects: resource access and child’s age. Controlling for the other factors, respondents judged that a hypothetical woman who is capable of feeding her children every day is almost a full step more likely to spend time with her offspring than a hypothetical woman who is not capable of doing so. Similarly, a hypothetical woman with a child who is 2-years old is approximately half a step less likely to spend time caring for her child than a hypothetical woman with a 3-month old. The other main and interaction effects of interest were neither substantively nor statistically significant.

Models Y_1_ and Y_2_ in Figure 2A were estimated using the younger and older halves of the sample respectively. The effects and statistical significances of resource access and child’s age are almost identical to those from the full model.

### Illness

**Figure 3:**
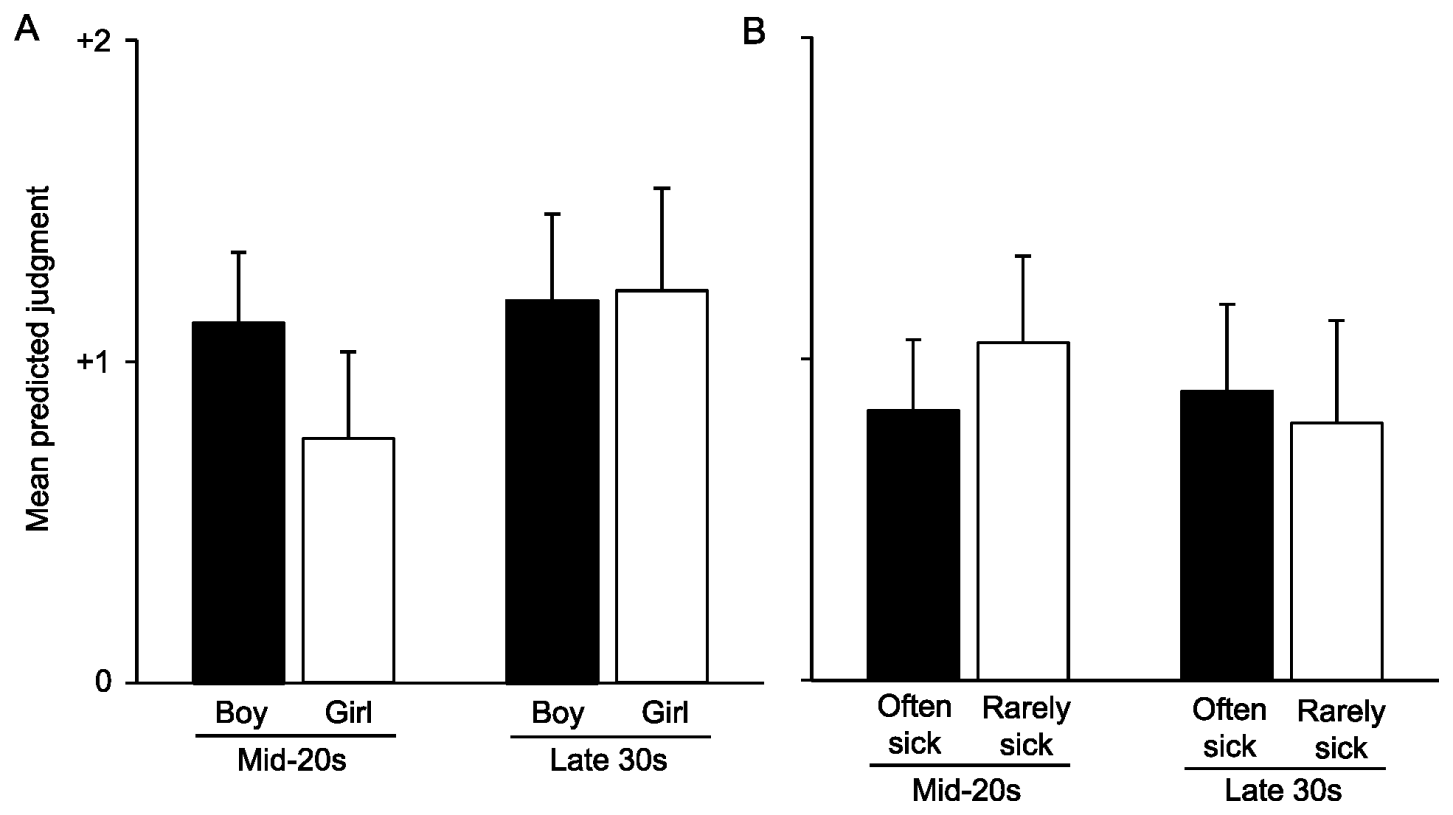
Interaction effects in the *Illness* model. Statistically significant interaction effects in the *Illness* model: (A) mother’s age x offspring gender, and (B) mother’s age x offspring viability. Error bars show the upper bounds of the 95% confidence interval.

I used a linear random-intercept model to estimate the effect of each factor and the interactions of interest on responses to the *Illness* question. This provided an adjustment for having multiple judgments per respondent. The significant coefficients are illustrated in Figure 2B. The full details of the models are included in the *SI* (see Table S5). Model X was estimated using all judgments from all respondents, and there were two substantively and statistically significant main effects: resource access and child’s gender. Controlling for the other factors, respondents judged that a hypothetical woman who is capable of feeding her children is exactly a full step more likely to seek medical care for her child than a hypothetical woman who is not capable of doing so. The estimate for child’s gender is best interpreted as an element of one of the two substantively and statistically significant interaction effects. In Figure 3A, hypothetical mothers in their mid-20s were judged to favor sons, whereas women in their late 30s were indifferent to offspring gender. In Figure 3B, hypothetical mothers in their mid-20s were judged to favor offspring who were rarely sick, whereas women in their late 30s were judged to be indifferent to offspring viability. The other main and interaction effects of interest were neither substantively nor statistically significant.

Models Y_1_ and Y_2_ in Figure 2B were estimated using the younger and older halves of the sample respectively. The effect of resource access, and the statistical significance of the coefficient, is remarkably similar in each of the two age models to the estimates in Model X. The effects for child’s gender, and the interaction of mother’s age and child’s viability, are almost identical across models, but are only statistically significant in the full model. The interaction of mother’s age and child’s gender are approximately the same, and are statistically significant in Model X and Y_1_.

## Discussion

In this study, 40 female respondents from two Karo Batak villages provided 640 judgments of the probable maternal-care decisions of 32 hypothetical women. A confounded factorial design allowed me to estimate the effects of five binary factors and their interactions with no confounding of the theoretically important effects for two distinct scenarios. In the Time question, the decision was whether to spend time with offspring if it came at the expense of economic productivity. In the *Illness* question, the decision was whether to incur a substantial expense of time and resources to get treatment for an ill child. For both, the basic hypothesis, derived from parental investment theory [17-19], was that natural selection will equip mothers with decision rules that optimize the tradeoff between investment in current and future offspring, or current offspring and other components of maternal fitness. Factors that increase fitness benefits will lead to increased care; factors that increased fitness costs will lead to decreased care. I summarize the results in Table 1 and discuss them below.

**Table 1:** Summary of evidence for decision rules based on the five factors of interest.

**Factor**	**Supported? ^a^**		**Effect**	**Decision Rule/Prediction^b^**	**Supported? ^c^**	
	***Time***	***Illness***			***Time***	***Illness***
**Mother’s Age:**	No	Yes	*Main:*	Increase care as you get older	No	No
			*Interaction:*	Increase care in daughters if you are young	No	No
				Increase care in sons if you are young	No	Yes
				Increase care in sickly offspring, but only if you are young	No	Yes
**Access to Resources:**	Yes	Yes	*Main:*	Increase care if you have good access to resources	Yes	Yes
			*Interaction:*	Increase care in sons if you have good access to resources	No	No
**Child’s Gender:**	No	Yes	*Interaction:*	Increase care in sons if you have good access to resources	No	No
				Increase care in daughters if you are young	No	No
				Increase care in sons if you are young	No	Yes
**Child’s Age:**	Yes	No	*Main:*	Increase care in younger offspring	Yes	No
				Increase care in older offspring	No	No
**Child’s Viability:**	No	Yes	*Main:*	Increase care if offspring is sickly	No	No
				Increase care if offspring is rarely sick	No	No
			*Interaction:*	Increase care in sickly offspring, but only if you are young	No	Yes

a Was there an effect of this factor in the full model, whether main or interaction?

b Predicted interaction effects are included twice each, once for each variable.

c Was there support for this decision rule/prediction specifically?

The factor with the strongest and most consistent effect was resource access, defined as a mother’s ability to provide food for her children. Food-secure mothers were judged as being approximately a full step more likely to provide care to offspring than food-insecure mothers in both questions and both young and old respondents. This conforms to prediction, and is consonant with theoretical and empirical findings (e.g., [1,30]) that mothers with better resource access pay a smaller opportunity cost for investing in current offspring. Food security is an apt measure of resource access for a number of reasons. First, it provides the study with better reproducibility because it translates well to any society, even technologically sparse ones [53]. Second, it is a strong predictor of maternal anxiety and depression [54], which might well be an element of the proximate mechanism driving this effect.

A handful of other factors had significant effects that were more-or-less consistent across models within questions, but not between them. In the Time question, mothers were judged as more likely to spend time with younger offspring. This conforms to prediction. Because younger offspring are more dependent, all else being equal, mothers get a larger dividend for providing them with care [17]. In the *Illness* question, there were two significant interaction effects that both conformed to prediction. As illustrated in Figure 3A, younger mothers were judged to favor sons while older mothers were indifferent. This is consonant with sex-allocation theory [55] and an adaptive strategy of the Karo Batak that I have documented elsewhere [40]. As illustrated in Figure 3B, younger mothers were judged to favor offspring that were rarely sick while older mothers were indifferent. With higher residual reproductive value, younger mothers are in a better position to produce a replacement offspring should the current one succumb to low viability. In sum, the decisions made in response to both scenarios take into account on a subset of the factors included in the study. The decision rule supported for the Time question included 2 of the 5 factors of interest; the decision rule for the *Illness* question included 4 of 5 (see Table 1). Because adaptations reflect a fit between form and function [56], the completeness of the decision rule for *Illness* may reflect a cost for imprecision given that there is more at stake in that scenario (i.e., the offspring has a realistic chance of death).

Some of the factors impinging upon the fitness costs and benefits of care appear to have no effect on the decision-making process in both scenarios (see Table 1). This suggests that a “simple heuristics” model of decision-making may be at play [57,58]. Under this model, natural selection equips organisms with simplified decision rules when the fitness payoffs of using them are better than rules that account for the full range of costs and benefits, or the acquisition of complete information that would allow one to make a better decision is too costly. To address the first point that simple rules are favored when they perform better, I highlight a mathematical model. Davis et al. [59] modeled avian offspring-provisioning strategies of varying complexity and found that simple rules—in particular, a decision rule that cued on environmental quality—outperformed more complex ones. This conforms to the results of this study that access to resources, which is shaped by environmental quality, is the only effect consistent across parental-care scenarios. To address the second point, I highlight the recognition by Clutton-Brock [17] that the complexity of the cost and benefit calculations for parental care would lead to strategies based on “simple rules of thumb.” Although there is considerable evidence that human parental strategies function to balance the trade-offs between various components of fitness, few studies have found a tight match between observed and predicted optimal strategies [2,3]. Kaplan and Lancaster [60] argue that a simple heuristic aimed at tracking diminishing returns on offspring investment may lead to lower than expected fertility in humans. In their discussion of optimality and human adaptation, El Mouden et al. [61] argue that heuristics are often favored by natural selection and can lead to solutions that appear suboptimal—e.g., if quantitative precision is unnecessary a “sundial” does as good a job as a “nuclear clock.”

Alternatively, the same results might be explained as an artifact of study design. First, the contrasts chosen for the binary factors might not reflect meaningful differences in the ecological context within which Karo Batak mothers care for offspring. The choice to use binary contrasts as independent variables was a constraint imposed by the factorial study design. The present design included 32 vignettes, representing every possible combination of levels for 5 binary factors. If I had added just a single additional level to each factor, the design would have bloated to 243 vignettes (i.e., 3^5^=243). Of course, offspring gender fits naturally into the binary scheme and for most of the others—resource access, child’s viability, and mother’s age—I used values approximating the extremes. Child’s age was an exception, but the contrast was strategic and meaningful. A 3-month old would likely still be breastfeeding, and a 24-month old is right on the cusp of weaning in Karo Batak society [42]. In the end, child’s age was part of the decision rule for the Time but not *Illness* question. We are left to wonder whether it would have been part of the rule for *Illness* if the contrast of child ages, and thus reproductive value, was more extreme. For instance, if the comparison was a 1-year old and a 10-year old, and that led to a significant effect for child’s age in the *Illness* model, then the decision rule would be optimal, not heuristic.

Second, respondents’ answers might reflect what they themselves, rather than the hypothetical mother, would do. This seems unlikely, however, as the judgments of young and old respondents were essentially identical for the Time question, and almost identical (though not all estimates were statistically significant) in the *Illness* question. The results reflect real-world maternal care decisions only in as much as the participants are adequate observers of the behavior of the real-world mothers around them. Although human maternal behavior is, to some degree, subject to hormonal and opioid influence, it is also strongly shaped by non-genetic factors such as culture [62,63]. A likely mechanism by which human mothers learn parenting skills is to stay “in-tune” to the behavior of other mothers which, for the purposes of this study, would mean that they would be likely to provide accurate judgments about third-party decisions. Further, some of the predicted interaction effects were found in the participants’ judgments, suggesting that the participants are skilled observers, perceptive with even the most subtle aspects of maternal decision-making.

Replication of the study in socioecologically diverse societies will be necessary to assess the universality of the decision rules documented here [64]. Further study also will address the shortcomings of this study. For instance, the questions posed to respondents varied in the amount of direct fitness at stake for the offspring, but in both cases the direct fitness cost to the parent was small. In further study, I suggest the inclusion of additional questions that vary the amount of direct fitness at stake for the mother. For instance, the hypothetical decision would put the mother’s life on the line rather than simply coming at the expense of economic productivity. This modification will allow for the testing of additional hypotheses about the strength, rather than just the direction, of various effects.

Vignette experiments have made only modest inroads to evolutionarily informed studies of human behavior. Judging from responses to the use of related methods (e.g., [65]), it is likely that ecological validity is a concern—i.e., whether decisions made in response to artificial scenarios reflect the sort of decisions that would be made in the real world. A number of studies have shown close matching of vignette responses and actual decisions [16]. As discussed above, some of the findings here are consonant with observations of Karo Batak parenting that I have documented elsewhere [40]. Although they stand far from usurping observation as the method of choice in human behavioral ecology, vignette experiments bring an experimental rigor that has been applied fruitfully to studies of non-human animal decision-making [8-10]. Further, vignettes allow for getting judgments about real-world phenomena that might not currently exist in the focal population. For these reasons, wider application in evolutionarily informed studies of human behavior is warranted.

## Supporting Information

Table S1 **Vignette studies published in *Evolution and Human Behavior* from 1997 to 2012.**
(DOCX)

Table S2 **Vignette sets.**
(DOCX)

Table S3 **Raw data.**
(DOCX)

Table S4 **Random-effects least-squares regression models for the Time question.**
(DOCX)

Table S5 **Random-effects least-squares regression models for the *Illness* question.**
(DOCX)
