# Supplementary material for: Access to Resources Shapes Maternal Decision Making: Evidence from a Factorial Vignette Experiment
Source: PLoS One. 2013 Sep 17;8(9):e75539. doi: 10.1371/journal.pone.0075539 (PMC3775810; doi:10.1371/journal.pone.0075539)
Supplement: Table S1 — Vignette studies published in Evolution and Human Behavior from 1997 to 2012. (DOCX) [file pone.0075539.s001.docx]

**Table S1.** Vignette studies published in *Evolution and Human Behavior* from 1997 to 2012.

| **Reference ^a^** | **Primary or Secondary Method** | **Study of Decision Rules? ^b^** | **Type of Design** | **Citation** |
| --- | --- | --- | --- | --- |
| Antfolk et al 2012 | Primary | No | Factorial | [1] |
| Bressler et al 2006 | Primary | No | Non-factorial | [2] |
| Brown & Lewis 2004 | Primary | No | Factorial | [3] |
| Fessler & Navarette 2004 | Primary | No | Non-factorial | [4] |
| Figueredo et al 2001 | Secondary | No | Non-factorial | [5] |
| Figueredo et al 2004 | Primary | Yes | Factorial | [6] |
| Kameda et al 2002 | Primary | Yes | Factorial | [7] |
| Kiyonari et al 2000 | Secondary | No | Factorial | [8] |
| Kurzban et al 2012 | Primary | Yes | Non-factorial | [9] |
| Lieberman & Lobel 2012 | Primary | No | Non-factorial | [10] |
| Mahalingam 2007 | Primary | Yes | Non-factorial | [11] |
| Ohtsubo & Watanabe 2009 | Primary | No | Non-factorial | [12] |
| Peterson et al 2012 | Primary | No | Factorial | [13] |
| Sheets et al 1997 | Primary | No | Non-factorial | [14] |
| Surbey & McNally 1997 | Primary | Yes | Factorial | [15] |
| Surbey 2011 | Primary | Yes | Factorial | [16] |

^a^ References found by searching for the word “vignette” in the title, abstract, keywords, and text of all 618 “Full-Length Articles” published in the journal *Evolution and Human Behavior* from 1997 to 2012. The search yielded 20 articles, but four were excluded because they only mentioned but did not use vignettes. My methodology may underestimate the prevalence of vignette studies in evolutionary social science if there are studies that used vignette methodologies but did not refer to them as such, or if evolutionary social scientists have preferentially published their vignette studies in other journals.

^b^ Studies of decision rules presented a vignette and asked the respondent about their own or a third-party’s behavior in response to the scenario; studies that did not cover decision rules presented a vignette and asked the respondent about psychological state.

References

1. Antfolk J, Karlsson M, Bäckström A, Santtila P (2012) Disgust elicited by third-party incest: the roles of biological relatedness, co-residence, and family relationship. Evol Hum Behav 33: 217-223.

2. Bressler ER, Martin RA, Balshine S (2006) Production and appreciation of humor as sexually selected traits. Evol Hum Behav 27: 121-130.

3. Brown SL, Lewis BP (2004) Relational dominance and mate-selection criteria: Evidence that males attend to female dominance. Evol Hum Behav 25: 406-415.

4. Fessler DMT, Navarrete CD (2004) Third-party attitudes toward sibling incest: Evidence for Westermarck's hypotheses. Evol Hum Behav 25: 277-294.

5. Figueredo AJ, Corral-Verdugo V, Frı́as-Armenta M, Bachar KJ, White J, et al. (2001) Blood, solidarity, status, and honor: The sexual balance of power and spousal abuse in Sonora, Mexico. Evol Hum Behav 22: 295-328.

6. Figueredo AJ, Tal IR, McNeil P, Guillén A (2004) Farmers, herders, and fishers: The ecology of revenge. Evol Hum Behav 25: 336-353.

7. Kameda T, Takezawa M, Tindale RS, Smith CM (2002) Social sharing and risk reduction: Exploring a computational algorithm for the psychology of windfall gains. Evol Hum Behav 23: 11-33.

8. Kiyonari T, Tanida S, Yamagishi T (2000) Social exchange and reciprocity: confusion or a heuristic? Evol Hum Behav 21: 411-427.

9. Kurzban R, DeScioli P, Fein D (2012) Hamilton vs. Kant: pitting adaptations for altruism against adaptations for moral judgment. Evol Hum Behav 33: 323-333.

10. Lieberman D, Lobel T (2012) Kinship on the Kibbutz: coresidence duration predicts altruism, personal sexual aversions and moral attitudes among communally reared peers. Evol Hum Behav 33: 26-34.

11. Mahalingam R (2007) Culture, ecology, and beliefs about gender in son preference caste groups. Evol Hum Behav 28: 319-329.

12. Ohtsubo Y, Watanabe E (2009) Do sincere apologies need to be costly? Test of a costly signaling model of apology. Evol Hum Behav 30: 114-123.

13. Petersen MB, Sell A, Tooby J, Cosmides L (2012) To punish or repair? Evolutionary psychology and lay intuitions about modern criminal justice. Evol Hum Behav 33: 682-695.

14. Sheets VL, Fredendall LL, Claypool HM (1997) Jealousy evocation, partner reassurance, and relationship stability: An exploration of the potential benefits of jealousy. Evol Hum Behav 18: 387-402.

15. Surbey MK, McNally JJ (1997) Self-deception as a mediator of cooperation and defection in varying social contexts described in the iterated prisoner's dilemma. Evol Hum Behav 18: 417-435.

16. Surbey MK (2011) Adaptive significance of low levels of self-deception and cooperation in depression. Evol Hum Behav 32: 29-40.
