# Supplementary material for: Access to Resources Shapes Maternal Decision Making: Evidence from a Factorial Vignette Experiment
Source: PLoS One. 2013 Sep 17;8(9):e75539. doi: 10.1371/journal.pone.0075539 (PMC3775810; doi:10.1371/journal.pone.0075539)
Supplement: Table S2 — Vignette sets. (DOCX) [file pone.0075539.s002.docx]

**Table S2.** Vignette sets.

| **Set** | **Vignette** | **Mother’s Age** | **Resource Access** | **Child’s Gender** | **Child’s Age** | **Child’s Viability** |
| --- | --- | --- | --- | --- | --- | --- |
| A | 1 | Mid 20's | Capable | Boy | 3 mo. | Sick often |
|  | 2 | Late 30's | Capable | Boy | 2 yrs. | Sick often |
|  | 3 | Mid 20's | Not capable | Girl | 2 yrs. | Rarely sick |
|  | 4 | Late 30's | Not capable | Girl | 3 mo. | Sick often |
|  | 5 | Mid 20's | Not capable | Boy | 2 yrs. | Sick often |
|  | 6 | Mid 20's | Not capable | Boy | 3 mo. | Rarely sick |
|  | 7 | Late 30's | Capable | Boy | 2 yrs. | Rarely sick |
|  | 8 | Late 30's | Not capable | Boy | 3 mo. | Rarely sick |
| B | 1 | Mid 20's | Capable | Girl | 2 yrs. | Sick often |
|  | 2 | Late 30's | Capable | Girl | 2 yrs. | Rarely sick |
|  | 3 | Late 30's | Not capable | Girl | 2 yrs. | Sick often |
|  | 4 | Mid 20's | Capable | Girl | 3 mo. | Rarely sick |
|  | 5 | Mid 20's | Not capable | Boy | 3 mo. | Sick often |
|  | 6 | Late 30's | Capable | Boy | 3 mo. | Sick often |
|  | 7 | Late 30's | Not capable | Boy | 2 yrs. | Rarely sick |
|  | 8 | Late 30's | Capable | Girl | 3 mo. | Sick often |
| C | 1 | Mid 20's | Not capable | Girl | 3 mo. | Sick often |
|  | 2 | Late 30's | Not capable | Boy | 2 yrs. | Sick often |
|  | 3 | Mid 20's | Capable | Girl | 2 yrs. | Rarely sick |
|  | 4 | Mid 20's | Capable | Boy | 2 yrs. | Sick often |
|  | 5 | Late 30's | Capable | Boy | 3 mo. | Rarely sick |
|  | 6 | Late 30's | Not capable | Girl | 3 mo. | Rarely sick |
|  | 7 | Mid 20's | Not capable | Boy | 2 yrs. | Rarely sick |
|  | 8 | Mid 20's | Capable | Girl | 3 mo. | Sick often |
| D | 1 | Late 30's | Capable | Girl | 3 mo. | Rarely sick |
|  | 2 | Mid 20's | Capable | Boy | 2 yrs. | Rarely sick |
|  | 3 | Mid 20's | Capable | Boy | 3 mo. | Rarely sick |
|  | 4 | Mid 20's | Not capable | Girl | 2 yrs. | Sick often |
|  | 5 | Late 30's | Capable | Girl | 2 yrs. | Sick often |
|  | 6 | Late 30's | Not capable | Girl | 2 yrs. | Rarely sick |
|  | 7 | Mid 20's | Not capable | Girl | 3 mo. | Rarely sick |
|  | 8 | Late 30's | Not capable | Boy | 3 mo. | Sick often |

**Table S2.** Vignette sets (cont.)

| **Set** | **Vignette** | **Mother’s Age** | **Resource Access** | **Child’s Gender** | **Child’s Age** | **Child’s Viability** |
| --- | --- | --- | --- | --- | --- | --- |
| E | 1 | Late 30's | Capable | Boy | 2 yrs. | Rarely sick |
|  | 2 | Mid 20's | Not capable | Boy | 3 mo. | Rarely sick |
|  | 3 | Late 30's | Not capable | Boy | 3 mo. | Rarely sick |
|  | 4 | Late 30's | Capable | Boy | 2 yrs. | Sick often |
|  | 5 | Mid 20's | Capable | Boy | 3 mo. | Sick often |
|  | 6 | Late 30's | Not capable | Girl | 3 mo. | Sick often |
|  | 7 | Mid 20's | Not capable | Girl | 2 yrs. | Rarely sick |
|  | 8 | Mid 20's | Not capable | Boy | 2 yrs. | Sick often |
| F | 1 | Late 30's | Not capable | Boy | 2 yrs. | Rarely sick |
|  | 2 | Late 30's | Capable | Boy | 3 mo. | Sick often |
|  | 3 | Late 30's | Capable | Girl | 3 mo. | Sick often |
|  | 4 | Late 30's | Capable | Girl | 2 yrs. | Rarely sick |
|  | 5 | Mid 20's | Capable | Girl | 2 yrs. | Sick often |
|  | 6 | Mid 20's | Capable | Girl | 3 mo. | Rarely sick |
|  | 7 | Late 30's | Not capable | Girl | 2 yrs. | Sick often |
|  | 8 | Mid 20's | Not capable | Boy | 3 mo. | Sick often |
| G | 1 | Mid 20's | Not capable | Boy | 2 yrs. | Rarely sick |
|  | 2 | Late 30's | Not capable | Girl | 3 mo. | Rarely sick |
|  | 3 | Mid 20's | Capable | Girl | 3 mo. | Sick often |
|  | 4 | Late 30's | Not capable | Boy | 2 yrs. | Sick often |
|  | 5 | Mid 20's | Not capable | Girl | 3 mo. | Sick often |
|  | 6 | Mid 20's | Capable | Boy | 2 yrs. | Sick often |
|  | 7 | Mid 20's | Capable | Girl | 2 yrs. | Rarely sick |
|  | 8 | Late 30's | Capable | Boy | 3 mo. | Rarely sick |
| H | 1 | Mid 20's | Not capable | Girl | 3 mo. | Rarely sick |
|  | 2 | Late 30's | Not capable | Girl | 2 yrs. | Rarely sick |
|  | 3 | Late 30's | Not capable | Boy | 3 mo. | Sick often |
|  | 4 | Mid 20's | Capable | Boy | 2 yrs. | Rarely sick |
|  | 5 | Late 30's | Capable | Girl | 3 mo. | Rarely sick |
|  | 6 | Mid 20's | Not capable | Girl | 2 yrs. | Sick often |
|  | 7 | Mid 20's | Capable | Boy | 3 mo. | Rarely sick |
|  | 8 | Late 30's | Capable | Girl | 2 yrs. | Sick often |
