# Supplementary material for: Access to Resources Shapes Maternal Decision Making: Evidence from a Factorial Vignette Experiment
Source: PLoS One. 2013 Sep 17;8(9):e75539. doi: 10.1371/journal.pone.0075539 (PMC3775810; doi:10.1371/journal.pone.0075539)
Supplement: Table S3 — Raw data. (DOCX) [file pone.0075539.s003.docx]

**Table S3.** Raw data.

|  |  |  |  | | **V1** | | **V2** | | **V3** | | **V4** | | **V5** | | **V6** | | **V7** | | **V8** | |
| --- | --- | --- | --- | --- | --- | --- | --- | --- | --- | --- | --- | --- | --- | --- | --- | --- | --- | --- | --- | --- |
| **Respondent** | **Set** | **Village** | **Age** | | **T** | **I** | **T** | **I** | **T** | **I** | **T** | **I** | **T** | **I** | **T** | **I** | **T** | **I** | **T** | **I** |
| 1 | A | Doulu | | 21 | 1 | 2 | 0 | 2 | 0 | 2 | 1 | 2 | 1 | 2 | 0 | 1 | 0 | 2 | 0 | 1 |
| 2 | B | Doulu | | 21 | 2 | 2 | 1 | 2 | 2 | 1 | 2 | 2 | 1 | 1 | 2 | 2 | 1 | 1 | 2 | 2 |
| 3 | C | Doulu | | 23 | 0 | 0 | 0 | 0 | 1 | 1 | 2 | 1 | 2 | 1 | 2 | 2 | 1 | 2 | 1 | 2 |
| 4 | D | Doulu | | 36 | 2 | 1 | 1 | 2 | 2 | 2 | 0 | 1 | 1 | 2 | 1 | 2 | 0 | 1 | 1 | 2 |
| 5 | E | Doulu | | 60 | 0 | 2 | 2 | 1 | 2 | 0 | 2 | 2 | 2 | 2 | 2 | 2 | 2 | 0 | 2 | 2 |
| 6 | F | Doulu | | 38 | 1 | 0 | 1 | 0 | 0 | 1 | 0 | 0 | 1 | 0 | 0 | 1 | 1 | 1 | 1 | 1 |
| 7 | G | Doulu | | 41 | -1 | 2 | 2 | 2 | 0 | 2 | 2 | 2 | 2 | 0 | 0 | 2 | 1 | 2 | 2 | -1 |
| 8 | H | Doulu | | 26 | 1 | 0 | -2 | 2 | -2 | 1 | 2 | 1 | 1 | 2 | 0 | 0 | 2 | 2 | 2 | 2 |
| 9 | A | Doulu | | 32 | 2 | 1 | 1 | 2 | -1 | 0 | 0 | 1 | -1 | -2 | 0 | 0 | 2 | 2 | 0 | -1 |
| 10 | B | Doulu | | 38 | 1 | 2 | 0 | 2 | -2 | -1 | 1 | 2 | 1 | 1 | 0 | 2 | 1 | 1 | 1 | 2 |
| 11 | C | Doulu | | 26 | 0 | 1 | 1 | 0 | 2 | 2 | 1 | 2 | 2 | 2 | -1 | 1 | 0 | 1 | 2 | 2 |
| 12 | D | Doulu | | 31 | 0 | 2 | 2 | 2 | 2 | 2 | 0 | 0 | 1 | 2 | 1 | 0 | 0 | 0 | 0 | 1 |
| 13 | E | Doulu | | 39 | 2 | 2 | 1 | 0 | 1 | 1 | 2 | 2 | 2 | 2 | 1 | 1 | 0 | 0 | -1 | 0 |
| 14 | F | Doulu | | 29 | -1 | 1 | 2 | 2 | 2 | 2 | 0 | 1 | 1 | 2 | 2 | 2 | -1 | 0 | -1 | 0 |
| 15 | G | Doulu | | 34 | -1 | 2 | 0 | 1 | 1 | 2 | 1 | 1 | 1 | 1 | 1 | 2 | 1 | 2 | 1 | 2 |
| 16 | H | Doulu | | 55 | 0 | -1 | 1 | -1 | 1 | -1 | 1 | 2 | 2 | 1 | -1 | -1 | 2 | 2 | 2 | 2 |
| 17 | A | Doulu | | 48 | 2 | 2 | 0 | 2 | -1 | 1 | -1 | 0 | -1 | 0 | 1 | 0 | 1 | 2 | -1 | 0 |
| 18 | B | Doulu | | 21 | 0 | 1 | 0 | 1 | 1 | 1 | 0 | 1 | 1 | 1 | 1 | 1 | 1 | 1 | 1 | 1 |
| 19 | C | Doulu | | 46 | 0 | 1 | 0 | 1 | 2 | 1 | 0 | 1 | 0 | 1 | 0 | 1 | 0 | 1 | 0 | 1 |
| 20 | D | Doulu | | 20 | 1 | 2 | 0 | 2 | 1 | 2 | -1 | -1 | 0 | 2 | -1 | -1 | 2 | -1 | 2 | -1 |

**Table S3.** Raw data (cont.)

|  |  |  |  | **V1** | | **V2** | | **V3** | | **V4** | | **V5** | | | **V6** | | | **V7** | | **V8** | |
| --- | --- | --- | --- | --- | --- | --- | --- | --- | --- | --- | --- | --- | --- | --- | --- | --- | --- | --- | --- | --- | --- |
| **Respondent** | **Set** | **Village** | **Age** | **T** | **I** | **T** | **I** | **T** | **I** | **T** | **I** | **T** | **I** | | **T** | **I** | | **T** | **I** | **T** | **I** |
| 21 | E | Laubuluh | 23 | 2 | 2 | -1 | 1 | -1 | 1 | 2 | 2 | 2 | 2 | -1 | | 1 | -1 | | 1 | -1 | 1 |
| 22 | F | Laubuluh | 26 | 0 | 1 | 2 | 2 | 0 | 2 | 0 | 2 | 0 | 2 | 2 | | 1 | 0 | | 1 | 1 | 1 |
| 23 | G | Laubuluh | 55 | -1 | 1 | 0 | -1 | 2 | 1 | 0 | 1 | -1 | -1 | 2 | | 1 | 1 | | 1 | 2 | 1 |
| 24 | H | Laubuluh | 37 | 2 | 1 | 0 | 2 | 2 | 1 | 1 | 2 | 2 | 2 | 0 | | -1 | 2 | | 1 | 0 | 1 |
| 25 | A | Laubuluh | 20 | 1 | 2 | 1 | 2 | 1 | 1 | 1 | 1 | 0 | 1 | 0 | | 1 | 0 | | 2 | 1 | 1 |
| 26 | B | Laubuluh | 20 | 0 | 2 | 0 | 2 | 1 | 1 | 0 | 1 | 1 | 1 | 0 | | 2 | 0 | | 1 | 0 | 1 |
| 27 | C | Laubuluh | 45 | 1 | -1 | 0 | 1 | 2 | 2 | 2 | 2 | 2 | 2 | -1 | | 0 | -1 | | 1 | 2 | 1 |
| 28 | D | Laubuluh | 20 | 1 | 2 | 0 | 2 | 1 | 2 | -1 | 0 | 0 | 1 | 0 | | 0 | 1 | | 0 | 0 | 1 |
| 29 | E | Laubuluh | 49 | 0 | 2 | 0 | 1 | 0 | 2 | 0 | 2 | 0 | 2 | 0 | | 1 | 0 | | 1 | 0 | 1 |
| 30 | F | Laubuluh | 32 | -1 | 0 | 0 | 2 | 0 | 2 | 0 | 0 | 0 | 0 | 2 | | 0 | 0 | | 1 | 2 | 0 |
| 31 | G | Laubuluh | 35 | -2 | 1 | 0 | 1 | 1 | 2 | -1 | 1 | 0 | 1 | 2 | | 2 | 1 | | 2 | 2 | 2 |
| 32 | H | Laubuluh | 48 | -1 | 1 | -1 | -2 | 0 | -1 | 0 | 1 | 2 | 0 | 0 | | -2 | 2 | | 1 | 0 | -1 |
| 33 | A | Laubuluh | 18 | 0 | -1 | 0 | -1 | 2 | -2 | 2 | 0 | 0 | -1 | 2 | | -1 | 0 | | 1 | 0 | -1 |
| 34 | B | Laubuluh | 26 | 0 | 2 | 1 | 2 | 1 | -1 | 0 | 1 | 0 | -1 | 0 | | 0 | 2 | | -1 | 0 | 1 |
| 35 | C | Laubuluh | 43 | 0 | 1 | 0 | 0 | 2 | 2 | 2 | 2 | 2 | 2 | 1 | | 1 | 1 | | 0 | 2 | 2 |
| 36 | D | Laubuluh | 39 | 2 | -1 | 0 | 1 | 0 | 2 | 1 | -1 | 0 | 2 | 0 | | 1 | 0 | | -1 | 2 | 1 |
| 37 | E | Laubuluh | 30 | 1 | 1 | 2 | 1 | 2 | 1 | 1 | 2 | 1 | 2 | 1 | | 1 | 1 | | 1 | 1 | 1 |
| 38 | F | Laubuluh | 46 | -1 | 1 | 1 | 1 | 1 | 2 | 1 | 2 | 0 | 2 | 1 | | 2 | 0 | | 0 | 0 | 0 |
| 39 | G | Laubuluh | 36 | 0 | 1 | 2 | 1 | -1 | 2 | -1 | 1 | 2 | 1 | 2 | | 2 | 1 | | 2 | 0 | 2 |
| 40 | H | Laubuluh | 48 | -1 | 1 | 0 | 1 | 0 | 1 | 2 | 1 | 2 | 2 | 1 | | 1 | 2 | | 1 | 2 | 1 |
