# Supplementary material for: Access to Resources Shapes Maternal Decision Making: Evidence from a Factorial Vignette Experiment
Source: PLoS One. 2013 Sep 17;8(9):e75539. doi: 10.1371/journal.pone.0075539 (PMC3775810; doi:10.1371/journal.pone.0075539)
Supplement: Table S4 — Random-effects least-squares regression models for the Time question. (DOCX) [file pone.0075539.s004.docx]

**Table S4.** Random-effects least-squares regression models for the *Time* question.

|  |  | **Model X (all ages)** | **Model Y_1_ (<35 y.o.)** | **Model Y_2_ (≥ 35 y.o.)** |
| --- | --- | --- | --- | --- |
| **Respondent:** | Age (yrs.) | <0.001 | -- | -- |
|  | Village | -0.163 | -0.163 | -0.310 |
| **Main effects:** | Mother’s age | -0.022 | 0.082 | -0.164 |
|  | Resource access | 0.831 *** | 0.788 *** | 0.833 *** |
|  | Child’s gender | -0.055 | 0.006 | -0.195 |
|  | Child’s age | -0.400 *** | -0.323 * | -0.440 ** |
|  | Child’s viability | 0.065 | 0.317 | -0.163 |
| **Interactions:** | Resource access x child’s gender | -0.178 | -0.401 | 0.102 |
|  | Mother’s age x child’s gender | 0.043 | 0.032 | 0.068 |
|  | Mother’s age x child’s viability | 0.688 | 0.229 | 0.331 |
| **Random effects:** | Sigma_u | 0.270 | 0.260 | 0.349 |
|  | Sigma_e | 0.906 | 0.907 | 0.906 |
| **Model fit:** | *n* (judgments) | 320 | 160 | 160 |
|  | *n* (respondents) | 40 | 20 | 20 |
|  | Constant | 0.688 | 0.229 | 1.052 |
|  | Wald χ^2^ | 77.61 *** | 33.38 ** | 55.45 ** |

*Notes:* Effects are unadjusted coefficients; * *p* < 0.05, ** *p* < 0.01, *** *p* < 0.001; models include set-effect adjustments.
