# Supplementary material for: Access to Resources Shapes Maternal Decision Making: Evidence from a Factorial Vignette Experiment
Source: PLoS One. 2013 Sep 17;8(9):e75539. doi: 10.1371/journal.pone.0075539 (PMC3775810; doi:10.1371/journal.pone.0075539)
Supplement: Table S5 — Random-effects least-squares regression models for the Illness question. (DOCX) [file pone.0075539.s005.docx]

**Table S5.** Random-effects least-squares regression models for the *Illness* question.

|  |  | **Model X (all ages)** | **Model Y_1_ (<35 y.o.)** | **Model Y_2_ (≥ 35 y.o.)** |
| --- | --- | --- | --- | --- |
| **Respondent:** | Age (yrs.) | -0.010 | -- | -- |
|  | Village | -0.214 | -0.356 | 0.035 |
| **Main effects:** | Mother’s age | 0.066 | -0.001 | 0.096 |
|  | Resource access | 0.995 *** | 1.087 *** | 0.910 *** |
|  | Child’s gender | -0.363 ** | -0.366 † | -0.382 † |
|  | Child’s age | 0.025 | -0.002 | 0.036 |
|  | Child’s viability | 0.219 † | 0.180 | 0.241 |
| **Interactions:** | Resource access x child’s gender | 0.103 | 0.041 | 0.147 |
|  | Mother’s age x child’s gender | 0.397 * | 0.540 ** | 0.254 |
|  | Mother’s age x child’s viability | -0.322 * | -0.211 | -0.364 |
| **Random effects:** | Sigma_u | 0.480 | 0.629 | 0.481 |
|  | Sigma_e | 0.688 | 0.625 | 0.756 |
| **Model fit:** | *n* (judgments) | 40 | 20 | 20 |
|  | *n* (respondents) | 320 | 160 | 160 |
|  | Constant | 0.691 | 0.646 | 0.630 |
|  | Wald χ^2^ | 204.63 *** | 132.80 *** | 80.63 *** |

*Notes:* Effects are unadjusted coefficients; * *p* < 0.05, ** *p* < 0.01, *** *p* < 0.001; models include set-effect adjustments.
